# Characterisation of rectal amoxicillin (RAMOX) for the treatment of pneumonia in children

**DOI:** 10.1007/s13346-020-00804-6

**Published:** 2020-06-25

**Authors:** Sara M Hanning, Silvia Matiz, Katharina Krasser, Mine Orlu, Cornelius Dodoo, Simon Gaisford, Catherine Tuleu

**Affiliations:** 1 UCL School of Pharmacy, University College London, 29-39 Brunswick Square, London, WC1N 1AX UK; 2 School of Pharmacy, The University of Auckland, 85 Park Road, Auckland, New Zealand; 3 School of Pharmacy, University of Health and Allied Sciences, Ho, Ghana

**Keywords:** Suppository, Amoxicillin, Rectal drug delivery, Paediatric, Formulation

## Abstract

Access to medicines, including their availability and affordability, is a major public health challenge worldwide. This research aimed to characterise rectal formulations containing amoxicillin for the treatment of pneumonia in children under five, as an accessible alternative to existing formulations. Lipophilic Suppocire (S-NA15) and hydrophilic polyethylene glycol (PEG; 80% PEG 1500 and 20% PEG 4000, w/w) suppositories containing 250 mg amoxicillin were prepared. Hardness, apparent viscosity, uniformity of mass, uniformity of content, disintegration and dissolution time were determined. Irritation potential was screened using a slug mucosal assay and antibacterial efficacy against *Staphylococcus aureus* determined by isothermal microcalorimetry. Both lipophilic and hydrophilic formulations met the European Pharmacopoeia standards for suppositories when tested in vitro. They disintegrated within 30 min with rapid amoxicillin release profiles (98.6 ± 0.9%, 94.9 ± 1.2% over 30 min, respectively). Over-encapsulation of S-NA15 suppositories with hydroxypropyl methylcellulose shells slowed drug release and improved stability over 2 months. S-NA15 suppositories were classified as non-irritant and PEG suppositories only mildly irritant. Antibacterial efficacy of formulations was equivalent to amoxicillin alone. Both PEG and over-encapsulated S-NA15 rectal formulations developed in the present work have shown promise based on pre-clinical screening, and further development is justified to develop a product with commercial potential.

## Introduction

Pneumonia is one of the leading causes of mortality globally in children under 5 years old [1], with an estimated 880,000 children dying of pneumonia in 2016 [2]. Pneumonia is caused by a variety of pathogens including bacteria, fungi and viruses—*Streptococcus pneumoniae* and *Haemophilus influenzae type B* are vaccine-preventable pathogens that are responsible for 18% and 7% of severe pneumonia episodes worldwide, respectively. Other pathogens have no available vaccine, for example, *Staphylococcus aureus* (*S. aureus*), *Salmonella*, *Klebsiella pneumoniae*, *Chlamydia pneumoniae* and *Mycoplasma tuberculosis* [3]; therefore, timely diagnosis and appropriate treatment are essential. Although effective tools exist for the prevention, diagnosis and treatment of pneumonia, these are not always accessible in the developing world, including countries situated in sub-Saharan Africa and South Asia, where the childhood pneumonia burden is high.

Amoxicillin is considered a “priority essential” medicine and is recommended by the World Health Organization (WHO) as first-line treatment for outpatient paediatric pneumonia [4–6]. However, child friendly dosage forms are not available in many countries, and adult formulations are often manipulated to achieve appropriate dosages, which can compromise efficacy, adherence and medicine stability [6]. Moreover, the adverse taste of antibiotics is an important consideration that affects compliance and drives up the cost of the medication, with the need for taste masking technologies. Capsules are the most widely used form of amoxicillin worldwide, but accurate dose manipulation is not possible, and paediatric patients often struggle to swallow them due to difficulties with coordination. For children, an oral suspension is often prescribed. This comes in a powder that is reconstituted with clean water, expiring approximately 2 weeks following reconstitution and requiring refrigeration during that time. However, oral liquids are not practical in many resource-poor countries due to a lack of clean water as well as paucity of refrigeration, transport and storage facilities. Further, there have been dosing errors in the past when administering the oral suspension form of amoxicillin [7]. This has led to the development of a dispersible amoxicillin tablet, which has more flexible storage requirements and has become the recommended dosage form for children in developing countries [6]. Like other oral dosage forms, administration is not possible in vomiting or unconscious children. In addition, dispersible tablets still require a small amount of liquid for effective administration, such as clean water or breast milk, which may not always be available.

Rectal drug delivery systems have been investigated previously in the management of diseases in resource-poor settings, including rectal artesunate for the treatment of malaria and azithromycin suppositories to treat bacterial infections [8–10]. The delivery of medicines via the rectal route has several advantages including low cost of manufacture, ease of administration without the need for medically trained carers, avoidance of taste and swallowability concerns associated with oral drug delivery as well as no need for clean water to safely administer the formulation [11]. Many countries with a high pneumonia burden have a hot climate, and, therefore, stability of rectal formulations at high temperatures is a key consideration. Suppository excipients generally comprise lipophilic fatty bases, which melt at the temperature of the rectum, or water-soluble bases, which dissolve in the rectum. Water-soluble bases can be formulated to melt at higher temperatures than lipophilic bases, which may be advantageous in a hot climate. Lipophilic bases, on the other hand, often comprise only one type of hard fat, so are considered optimal for paediatrics in terms of minimising the additive risk associated with using multiple excipients [11]. A recently published study on doxycycline suppositories found that both lipophilic cocoa butter and hydrophilic polyethylene glycol (PEG 4000 and 400) formulations offered comparable bioavailability following rectal administration to rabbits. Further, plasma concentrations remained over the minimum inhibitory concentration (MIC) for more than 5 hours after administration [12]. In contrast, a study investigating rectal gentamicin in cocoa butter suppositories and liquid enemas found that gentamicin was not absorbed from the rectum in a minipig model, possibly due to the high polarity of the gentamicin molecule [13]. In the present study, the properties of both hydrophilic and lipophilic amoxicillin suppositories were explored, using commercially available bases. The target product profile included use of established commercial excipients with known safety profile, low cost, precedent of use in paediatric products, rapid disintegration and complete immediate release to ensure availability of a full and reproducible dose, and minimum irritation potential to improve patient acceptability. Stability at high temperatures was desirable in case refrigeration of formulations was not possible.

Lipophilic suppositories are formulated to melt at body temperature (around 37 °C), forming a thin film across the rectal mucosa and allowing the drug to diffuse out and then be absorbed. Hard fats are the most common lipophilic suppository base. The two main characteristics of hard fats are their drop point (32 °C to 45 °C) and hydroxyl value (3 to 50 mg KOH per gram of fat). The hydroxyl value influences the potential reactivity of the base. If it is high, the base can easily adsorb large amounts of water, which may be less appropriate for formulations with easily hydrolysing compounds [14]. As amoxicillin trihydrate is susceptible to hydrolysis [15], a lipophilic base with a low hydroxyl value may be preferred. A previous study compared different fatty bases in the formulation of amoxicillin suppositories and found that drug release from bases with hydroxyl values between 5 and 15 was greater and more complete than release from bases with a higher hydroxyl value [16]. Suppocire NA-15 (S-NA15) is a semi-synthetic glyceride base comprising saturated C10–C18 triglycerides. It was selected for the present study based its hydroxyl value of 12, as well as its performance in preliminary screening tests.

Hydrophilic bases are designed to dissolve in the 1–3 mL available rectal fluid to facilitate drug release. They have higher melting points and, therefore, may be better suited to high-temperature environments. A previous study by Kauss and colleagues addressed this in the development of azithromycin suppositories, where stability in tropical conditions was included as part of the target product profile [9]. It was hypothesised that by selecting excipients with high melting points (above 50 °C), stability at high temperatures would be improved, so high-molecular weight PEG was chosen as water-soluble suppository base. However, PEG is known to be slightly irritant to the rectal mucosa due to its hygroscopic nature, which may limit acceptability. Preclinical assessment of the irritation potential of rectal formulations often involves animal models [17, 18]. However, it would be useful to have an in vitro screening tool to reduce the number of animals needed for pharmaceutical research. One in vitro method with possible application to rectal formulations is a slug mucosal irritation assay. Slugs that are placed on an irritating substance will produce mucus. Further, if proteins and enzymes are released from the mucosal surface, then this suggests tissue damage. A classification prediction model has been developed that distinguishes between irritation (mucus production) and tissue damage (release of proteins) [19, 20]. In the present study, a PEG mixture of 80% w/w PEG 1500 and 20% w/w PEG 4000 was selected as hydrophilic base as it has a higher melting point of 45 °C and has been successfully used in the formulation of azithromycin suppositories [9, 21].

The aim of this project was to formulate and compare hydrophilic (PEG) and lipophilic (S-NA15) rectal formulations containing 250 mg amoxicillin. Affordable suppositories with stability in high-temperature environments were desired for their potential application in the treatment of pneumonia in children.

## Materials and methods

### Materials

Amoxicillin trihydrate (amoxTH) was purchased from Alfa Aesar (Lutterworth, UK). Novata B was kindly provided by BTC-Europe (Cheadle Hulme, UK). Suppocire NA15 (S-NA15) was a gift from Gattefossè SAS (St. Priest, France). Hydroxypropyl methylcellulose (HPMC) capsules (VCaps®, size 000) were gift samples from Capsugel (Strasbourg, France). PEG 1500 and 4000, water for HPLC, acetonitrile, orthophosphoric acid, Dulbecco A phosphate-buffered saline (PBS) tablets (sodium chloride 8.0 g/L, potassium chloride 0.2 g/L, disodium hydrogen phosphate 1.15 g/L, potassium dihydrogen phosphate 0.2 g/L), nutrient broth and nutrient agar were purchased from Fisher Scientific (Loughborough, UK). Commercial 250 mg amoxicillin oral capsules were purchased from AAH Pharmaceuticals (Coventry, England, UK). All chemicals and excipients were of pharmaceutical or analytical grade.

### Preparation of suppositories

Formulations of 250 mg amoxicillin (287 mg amoxTH) comprising either a lipophilic base (S-NA15) or a hydrophilic base (PEG mixture of 80% w/w PEG 1500 and 20% w/w PEG 4000) were developed. Suppositories were prepared using the moulding method for suppository preparation. Batches of 30 projectile-shaped suppositories were manufactured by melting each base in a water bath at 40 °C for S-NA15 formulations or 80 °C for PEG formulations. Simultaneously, amoxTH powder was ground using mortar and pestle. Once the base had fully melted, amoxTH was dispersed by mechanical stirring at 400 rpm before being transferred into a 1 g stainless steel 30-well suppository mould (Heros Ltd., Olomouc, Czech Republic). Suppositories were left to solidify for at least 45 min at room temperature before being carefully removed from the mould and stored at 4 °C until further use. In order to counteract melting at high temperatures, some S-NA15 suppositories were over-encapsulated into size 000 HPMC capsules (S-NA15_OEC_). In addition to the medicated batches, a batch of non-medicated suppositories were prepared for each suppository base and used as blank controls for the analytical tests.

### Compatibility of amoxicillin suppositories

#### FT-IR spectroscopy

Fourier transform infrared (FTIR) spectroscopy was carried out using a Spectrum 100 FTIR spectrometer (PerkinElmer, Waltham, MA, USA) in transmission mode over a spectral range of 4000–650 cm^−1^. Samples analysed included amoxTH, S-NA15, a PEG mixture comprising 80% w/w PEG 1500 and 20% w/w PEG 4000 and medicated S-NA15 and PEG suppositories.

#### Thermogravimetric analysis

Thermal stability of samples of amoxTH, S-NA15 and a PEG mixture of 80% w/w PEG 1500 and 20% w/w PEG 4000 were evaluated via thermogravimetric analysis (TGA), using a Discovery TGA instrument (TA Instruments, New Castle, DE, USA). Precisely weighed samples of 3–5 mg were placed into a Tzero aluminium pan and heated from 0 to 400 °C at a ramp rate of 2 °C min^−1^ under a flow of nitrogen N_2_ (50 mL min^−1^). Data analysis was conducted using the TA Universal Analysis software.

#### Differential scanning calorimetry

Differential scanning calorimetry (DSC) analyses were conducted using a Q2000 differential scanning calorimeter (TA Instruments, New Castle, DE, USA) with TA Universal Analysis software. AmoxTH, each of the suppository bases (S-NA15; 80% PEG 1500 + 20% PEG 4000), blank and medicated suppositories, as well as a physical blend of amoxTH and base, whose ratio was the same as that of the corresponding medicated suppository, were each tested in triplicate. In order to obtain a homogenous blend of amoxTH and base, excipients were ground using mortar and pestle prior to analysis. Precisely weighed samples (few mg) were placed in aluminium pans before being sealed with a pin-holed lid. An empty sealed aluminium pan covered with a perforated lid was used as a reference. A heating rate of 2 °C min^−1^ in the range from 0 to 230 °C was applied under a 50 mL min^−1^ flow of nitrogen gas.

### HPLC analysis of amoxicillin

An HPLC method was developed in accordance with a previous study on the analysis of amoxicillin and its impurities [22]. Chromatographic experiments were carried out using both Agilent 1220 LC System and Agilent 1260 Infinity (Agilent technologies, Santa Clara, USA). Samples were eluted through a Synergi 4 μm Polar-RP 80 Å, 250 × 4.6 mm column (Phenomenex, Macclesfield, UK) at 40 °C. Mobile phases comprised 20 mM potassium dihydrogen orthophosphate buffer (pH 4.8) and acetonitrile, whose gradient elution is described in Table 1. Samples (10 μL) were injected and eluted at a flow rate of 1.0 mL/min, and amoxTH was detected at a wavelength of 210 nm. Concentration of amoxTH (mg/mL) was then extrapolated as a function of the area of the peak (mAU*s), using a calibration curve covering a range from 0.0 to 1.4 mg/mL (*R*^2^ = 0.9914 for Agilent 1220 LC System; *R*^2^ = 0.9981 for Agilent 1260 Infinity).

**Table 1:** Gradient elution for HPLC analysis of amoxicillin: concentration of the two mobile phases over time

Time (min)	Potassium dihydrogen orthophosphate buffer (% v/v)	Acetonitrile (% v/v)
0:00	95	5
6:00	95	5
20:00	40	60
20:50	95	5
27:00	95	5

### In vitro characterisation of amoxicillin formulations

Suppositories were assessed visually for clarity (clear/opaque), surface texture (smooth/rough), appearance (dry/oily), tackiness, cracking and discolouration using a 5-point scale similar to that reported previously [23]. Where possible, European Pharmacopoeia (Ph. Eur.) guidelines were used in the characterisation of suppositories [24]. For uniformity of mass, twenty randomly selected suppositories from each batch were individually weighed. The mean, standard deviation and percentage deviation were calculated. In order to pass the uniformity of mass test, no more than two suppositories may have a percentage deviation over 5%, and none may deviate by more than 10% of the mean mass. Uniformity of content was evaluated from ten randomly selected suppositories per batch. Each suppository was weighed, placed into a beaker containing 250 mL water and mixed for 35 min under agitation at 300 rpm. PEG suppositories were left to dissolve at room temperature, whereas lipophilic suppositories were melted in a water bath at 55 °C then placed in a cold-water bath for the fatty bases to solidify. Samples of 2 mL were taken from the hydrophilic phase of each preparation, filtered with a 0.45-μm filter and analysed via HPLC as described above. Uniformity of content test was considered acceptable if not more than one content of amoxTH was outside the limits of 85 to 115% of the mean amoxTH content, and all samples were within the limits of 75 to 125% of the average amoxTH content.

#### Hardness

The minimum mechanical force required to break the suppositories (*n* = 10/batch) was determined using a tablet hardness tester (Copley Scientific Ltd., Nottingham, UK). Each suppository was oriented the same way such that the apex was pointed up and the longest side was parallel with the jaws.

#### Apparent viscosity

A Bohlin Gemini HR Nano rotational rheometer system (Malvern Instruments Ltd., Malvern, UK) was used to measure apparent viscosity of molten suppositories, both blank and medicated (*n* = 3/batch), using a 40-mm parallel plate geometry. Samples of approximately 1 mL were placed onto the lower plate, and the gap width was adjusted to 400 μm. Measurements were taken in a sweep time of 5 min with a shear rate range increasing from 1 to 200 s^−1^. Lipophilic suppositories were melted at 50 °C, applied to the lower plate at 37 °C and tested. When studying the rheology of S-NA15_OEC_, the HPMC shell was removed, and only its content was melted and analysed, as the study was focused on the rheological behaviour of fatty base S-NA15, in which amoxTH had been previously dispersed. Determining viscosity of hydrophilic suppositories was more challenging because of their high melting point, so two methods were investigated. First, the formulation was melted at 70 °C before being applied to the lower plate heated to 70 °C. Second, to mimic dilution in rectal fluids, the formulation was dissolved in 3 mL deionised water before being applied to the parallel plate and tested at 37 °C, introducing a dilution factor compared with the other methods.

#### Disintegration

To assess whether the suppositories softened or dissolved within the limits imposed by the International Pharmacopoeia (Ph. Int) [25], corresponding to 30 min for lipophilic suppositories and 60 min for the hydrophilic ones, a suppository (*n* = 3/batch) was placed into a basket then lowered into 250 mL deionised water at 37 ± 0.2 °C. Time taken for disintegration was recorded.

**Fig. 1:**
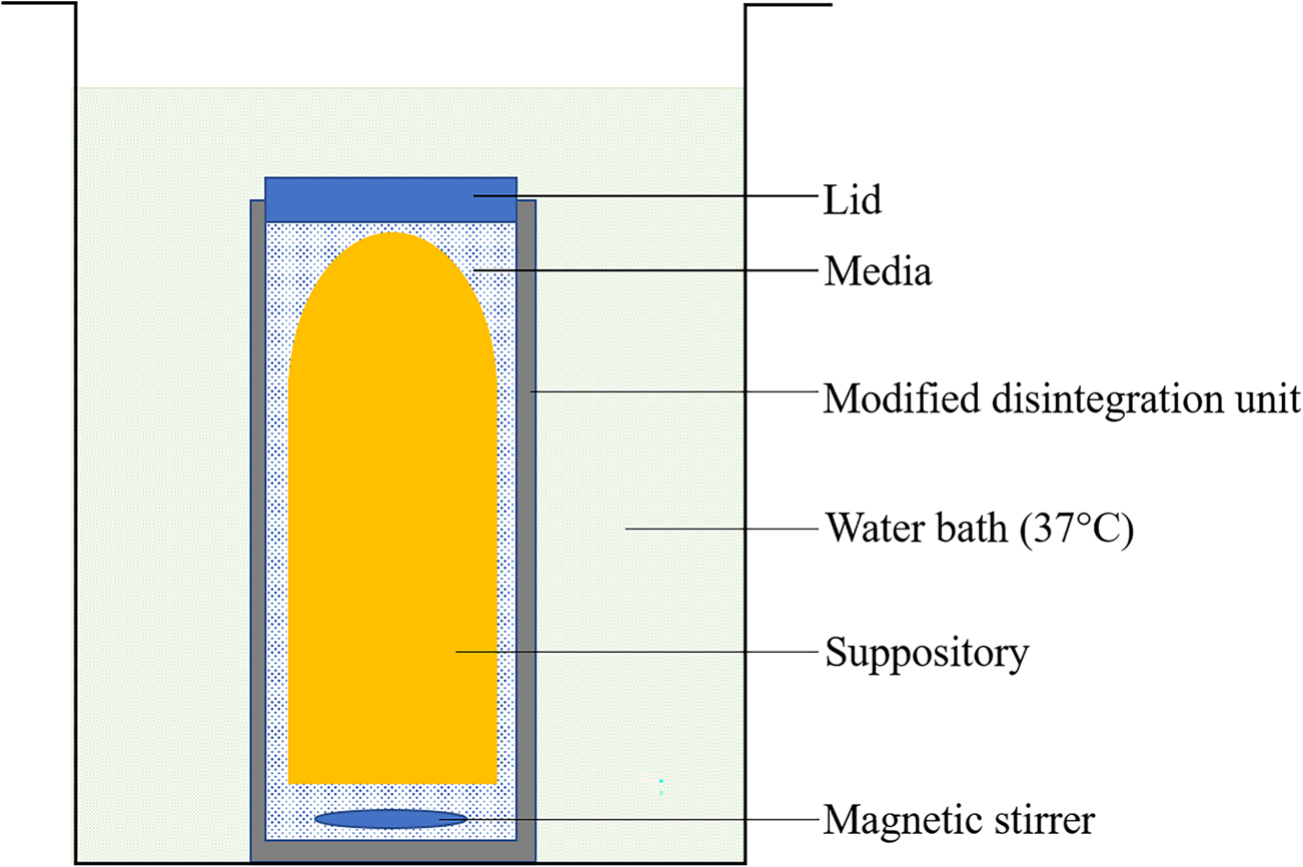
Visual representation of the modified disintegration test set-up, where the unit containing the suppository and 3-mL media was placed in a 37 °C water bath

An adapted disintegration test was also developed to better reflect the rectal environment (Fig. 1). A small custom-made lidded unit was created in-house to hold a small magnetic flea whose speed was set at 100 rpm, a suppository and 3 mL deionised water and then placed in a 37 °C water bath. The time taken for the medicated suppository (*n* = 3/batch) to completely melt (S-NA15-based formulations) or dissolve (PEG-based formulations) was recorded.

#### Dissolution

Dissolution tests were performed using Ph. Eur. Apparatus I (basket apparatus) [24], with a similar method to that reported in a previous study [9]. Briefly, each vessel contained 250 mL PBS (pH 7.3 ± 0.2) as the dissolution medium. Suppositories (*n* = 3/batch) were placed into baskets within the dissolution medium and rotated at 75 rpm. The temperature was set at 37 ± 0.2 °C. Samples of 2 mL were manually withdrawn from each basket at 0, 15, 30, 45, 60, 80, 100 and 120 min and then replaced with fresh media at the same pH and temperature conditions. Withdrawn samples were filtered with a 0.45 μm Millex® syringe filter and analysed via the HPLC method described above. Dissolution profiles were then compared with that of commercial oral 250 mg amoxicillin capsules (*n* = 3), whose drug release profile was evaluated under the same conditions.

### Irritation potential

The stinging, itching or burning potency of formulations were screened using a slug mucosal irritation assay. Suppositories formulated with PEG 1500/4000 and S-NA15 (both blank and medicated with amoxTH) and amoxTH alone were evaluated by placing slugs (*Arion lusitanicus*; *n* = 3 per treatment group) on 10 mg of each test item for 30 min daily over five successive days. Based on previous research, Novata B (a glyceride base mainly comprising C12–C14 glycerides) was used as the negative control, and Novata B with 10% sodium lauryl sulphate (SLS) was the positive control [26, 27]. The amount of mucus produced was expressed as percentage of body weight before the contact period. The irritation potency of the test formulation was then estimated using a previously developed classification prediction model [28]. Briefly, total mucus production for the five test days (% body weight) of < 7% was classed as non-irritant, 7–11.9% mildly irritant, 12–19.9% moderately irritant and 20% or greater was classed as severely irritant.

### Antibacterial efficacy against *S. aureus*

Antibacterial activity of both S-NA15 and PEG formulations against *S. aureus* was assessed with plate counts and isothermal microcalorimetry (IMC) using a previously described method by Said and colleagues [29]. Briefly, the challenge organism (*S. aureus*) was grown overnight in nutrient broth at 37 °C for 16 h before being harvested. This was then washed in PBS and resuspended in 15% v/v glycerol in Ringer’s solution. Aliquots (1 mL), with an average bacterial density of 10^6^ colony-forming units (CFU)/mL, were then immersed in liquid nitrogen until frozen and stored at − 80 °C until further use. All experiments were conducted under aseptic conditions. For each analysis, a frozen aliquot of *S. aureus* was immersed in a water bath at 40 °C for 3 min and then vortexed for 10 s. After each thawing, stock uniformity was determined and checked for any contamination. Pre-warmed nutrient broth (14.85 mL) was pipetted into a sterile 20-mL glass calorimetric ampoule, and 150 μL of the 10^6^ CFU/mL bacterial suspension was added. The ampoule was then hermetically sealed with a crimper, vortexed for 10 s and placed into a 2277 Thermal Activity Monitor (TA Instruments Ltd., UK) set at 37 °C to reach thermal equilibrium. After 30 min post-bacterial inoculation, data capture was initiated over 20 h using the dedicated software package Digitam 4.1. Once removed from the calorimeter, the population of *S. aureus* was determined using the serial dilution method and colony plate counts. Medicated suppositories containing 1.920 mg amoxTH and either S-NA15 of PEG were prepared and transferred into ampoules containing 15 mL nutrient broth (comprising 150 μL of 10^6^ CFU/mL). Ampoules were sealed, vortexed and analysed as described above. Finally, the antibacterial efficacy of 1.920 mg pure amoxTH against *S. aureus* was determined in triplicate as above.

### Preliminary stability studies

Preliminary stability studies were conducted on both lipophilic and hydrophilic formulations over 2 months under refrigeration (4 °C), ambient conditions (23 °C/40% relative humidity, RH), accelerated storage conditions (40 °C ± 2 °C, 75% RH ± 5% RH) [30] and 40 °C/10% RH. Each formulation was placed into a glass vial, able to contain any melting, as well as any possible leakage of content in the capsule-based formulations. Vials were placed into aluminium pouches with ziplock and stored at 4 °C and ambient conditions. Formulations for storage at 40 °C/75% RH and 40 °C/10% RH were placed into sealed aluminium pouches whose internal atmosphere had been previously modified with nitrogen flow, to improve stability of the formulations against the harsh storage conditions.

## Results and discussion

### Compatibility of amoxicillin suppositories

The DSC thermograms of pure amoxTH samples showed an endothermic peak at a temperature range between 64.6 and 88.8 °C, corresponding to dehydration of amoxTH to its anhydrous form. A second endothermic peak was observed at temperatures higher than 180 °C, corresponding to the initial decomposition of the anhydrous amoxicillin in the solid state. Fatty base S-NA15 started melting at a temperature range between 33 and 35 °C, whereas the hydrophilic physical mixture of 80% w/w PEG 1500 and 20% w/w PEG 4000 resulted in two peaks at 48.5 °C and 57.3 °C, corresponding to PEG 1500 and PEG 4000, respectively. The melting points of both fatty base (S-NA15) and hydrophilic (PEG mixture) base were not affected by the presence of amoxTH. However, in both cases, the process of dehydration of amoxTH was more difficult to detect, compared with the thermograms of pure amoxTH. The area corresponding to the melting process of amoxTH followed by degradation was also lower compared with a pure sample of amoxTH, as the base acted like an impurity for the drug. The DSC traces of blank suppositories were different than the corresponding base alone, as a result of the manufacturing process of suppositories. When testing medicated suppositories, the peak corresponding to the dehydration of amoxicillin shifted to higher temperatures compared with that of the pure drug. This could be related to the homogeneity of amoxTH dispersed within the base, which inevitably prolonged the dehydration process of amoxTH to its anhydrous form. No extra peaks were recorded in the DSC traces of medicated formulations, suggesting that no interactions between drug and base occurred. This hypothesis was also confirmed by comparing the FT-IR spectra of a pure sample of amoxTH with those of medicated suppositories. The β-lactam ring, responsible for the antibacterial activity of amoxicillin, absorbs at a frequency of 1776 cm^−1^. This peak was recorded in a pure sample of amoxTH as well as in medicated suppositories, indicating no incompatibilities between amoxTH and either the lipophilic or hydrophilic base. While an interaction between PEGs and the beta-lactam ring of penicillin antibiotics such as amoxicillin has been reported previously, this may not be clinically relevant [31] and was not observed in the present study.

### In vitro characterisation of amoxicillin suppositories

All suppositories had an off-white, opaque appearance when freshly manufactured. S-NA15 suppositories were oily and smooth whereas PEG suppositories appeared drier, rough and slightly stickier than the lipophilic ones. No suppositories presented cracks, indicating an adequate cooling process during manufacture, and there was no discolouration observed. Discolouration could arise if the viscosity of the base was too low, leading to an inhomogeneous distribution of amoxTH throughout the suppository mass. The uniformity of mass, uniformity of content and disintegration time of both lipophilic and hydrophilic formulations are summarised in Table 2.

**Table 2:** Summary of attributes of lipophilic (S-NA15 and S-NA15_OEC_) suppositories and hydrophilic (PEG, comprising 80% w/w PEG 1500 and 20% w/w PEG 4000) suppositories

Test		S-NA15	S-NA15_OEC_*	PEG**
Uniformity of mass, *n* = 20	Mean weight (g) (± SD)	1.11 (0.01)	1.27 (0.01)	1.31 (0.01)
	Mean deviation %	0.52	0.61	0.85
	Ph. Eur. Pass/fail^A^	**Pass**	**Pass**	**Pass**
Uniformity of content, *n* = 10	Mean content % (±SD)	102.1 (9.6)	99.1 (2.8)	95.4 (3.1)
	Ph. Eur. Pass/fail^B^	**Pass**	**Pass**	**Pass**
Mechanical strength, *n* = 10	Average force, *N* (± SD)	160.4 (28.3)	NT	306 (29.5)
Disintegration, *n* = 3	Standard test mean time, min (± SD)	5.3 (0.4)	NT	12.2 (1.4)
	Adapted test mean time, min (± SD)	16.3 (3.5)	23.0 (2.5)	12.1 (1.2)
	**Ph. Int Pass/fail**^**C**^	**Pass**	**Pass**	**Pass**

Each formulation contained 250 mg amoxicillin. Unless otherwise stated, values are presented as mean (SD). NT not tested

**Fig. 2:**
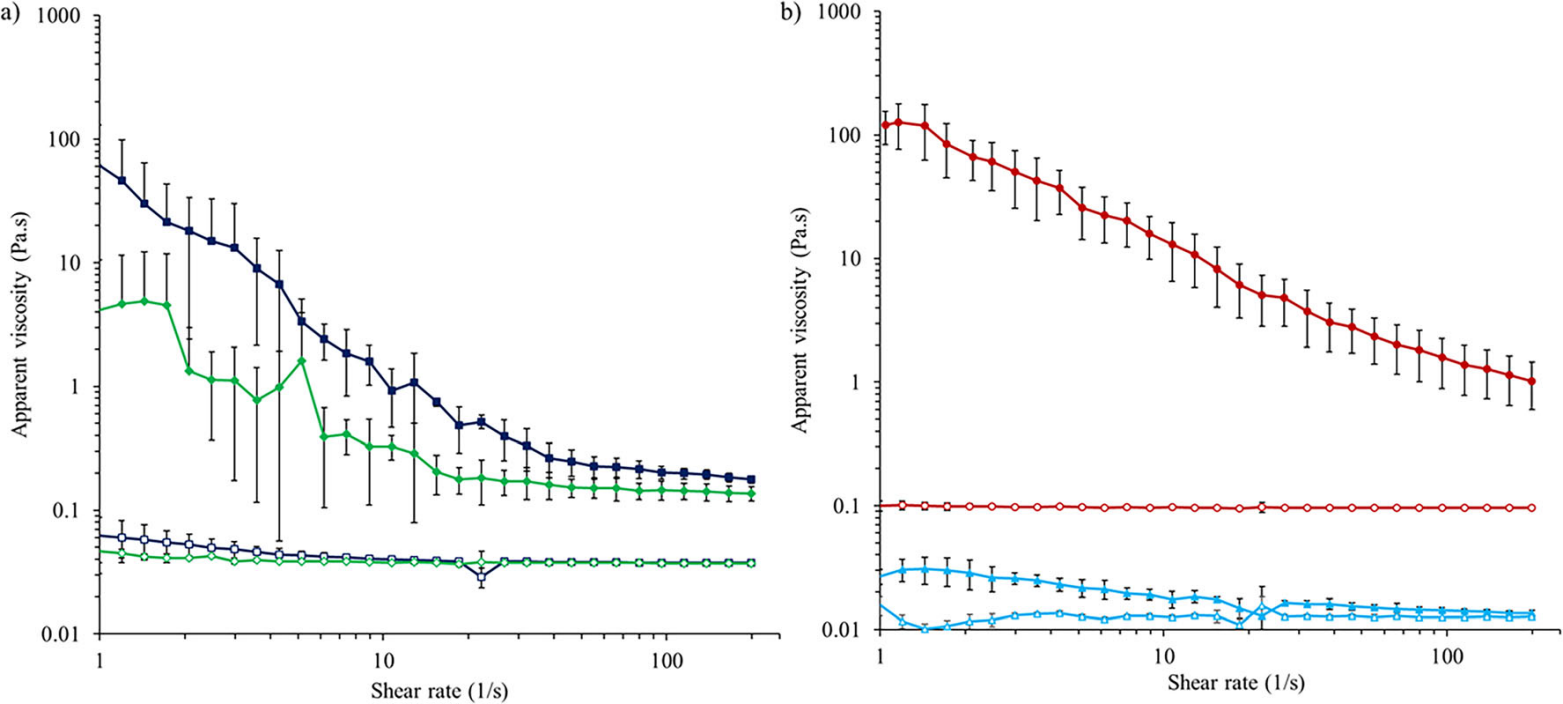
Apparent viscosity (*n* = 3; mean ± SD) as a function of shear rate for **a** lipophilic suppositories at 37 °C comprising S-NA15 base (green diamond) or S-NA15 base in an HPMC capsule (dark blue square), **b** PEG suppositories melted at 70 °C (red circle) or dissolved in 3 mL water (light blue triangle). Filled symbols denote suppositories medicated with 250 mg amoxicillin; unfilled symbols denote unmedicated (blank) suppositories

All formulations passed the tests of uniformity of mass and content, according to the limits imposed by the Ph. Eur. Evaluation of the mechanical strength of medicated suppositories through the crushing test showed that a higher force was required to break PEG suppositories (306 ± 29.5 N) compared with S-NA15 suppositories (160.4 ± 28.3 N), which may be advantageous for stability during storage and transport. All formulations were characterised by a low apparent viscosity, although it is difficult to compare the hydrophilic and lipophilic formulations due to the different melting temperatures. Blank suppositories showed that both lipophilic base S-NA15 and hydrophilic PEG mixture (80% w/w PEG 1500 and 20% w/w PEG 4000) behaved like Newtonian fluids, with apparent viscosity independent of shear rate (Fig. 2). Unsurprisingly, medicated suppositories were more viscous than the respective blank ones, due to the presence of amoxTH dispersed in the base. Variability within medicated formulations in the rheological measurements was observed, which could be related to the distribution of amoxTH within the base. As expected, dissolution of PEG suppositories in 3 mL deionised water prior to rheological measurement resulted in a decrease in apparent viscosity compared with PEG suppositories melted at 70 °C. This was due to dilution of the samples in water but may represent the apparent viscosity of the suppository as it melts in rectal fluid. In vivo, apparent viscosity is important because if this is too low, drug may leak from the rectum before being absorbed and if it is too high, spreading over the entire rectal mucosa may not occur, which could limit absorption.

Lipophilic formulations disintegrated within 30 min, and PEG suppositories disintegrated within 60 min under both standard and adapted conditions, as required by the Ph. Int for lipophilic and hydrophilic formulations. The disintegration process was deemed complete when all components had completely melted or dissolved, depending on their physicochemical properties. As expected, the disintegration time of S-NA15_OEC_ was longer than that of non-encapsulated S-NA15 suppositories (23.0 ± 2.5 min compared with 16.3 ± 3.5 min for the adapted test), as the HPMC outer shells had to disintegrate before the content could be released. For comparison, the disintegration time for the commercial oral capsules was 8.4 ± 1.5 min using the adapted test, which was nearly 4 min faster than the PEG suppositories. In some cases, parts of the HPMC shell stuck to the inner wall of the device, becoming gelatinous which delayed dissolution. This behaviour could affect rectal absorption of amoxTH in vivo or cause irritation of the rectal mucosa. Recent research has supported the concept of rectal capsules by successfully developing rectal ceftriaxone capsules for neonates in developing countries [32]. However, gelatin capsules were used due to the faster disintegration times compared with HPMC. This was not investigated in the present study as cultural or religious reasons may preclude the use of gelatin in some individuals [11, 33].

**Fig. 3:**
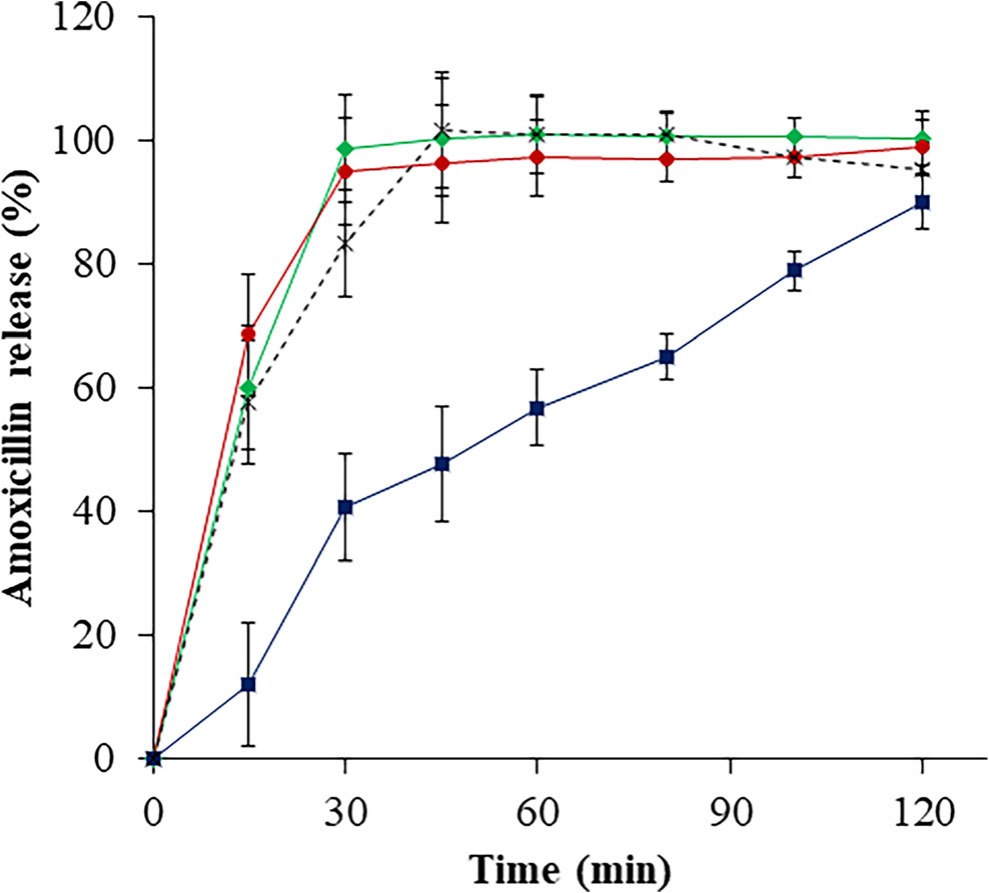
Drug release profile (mean ± SD; *n* = 3) of amoxicillin from S-NA15 suppositories (green diamond), PEG suppositories (red circle), S-NA15_OEC_ (S-NA15 suppositories over-encapsulated with HPMC shells) (dark blue square), commercial amoxicillin oral capsules (dotted line)

When testing dissolution under Ph. Eur. conditions, both S-NA15 and PEG suppositories offered the fastest and most complete drug release profiles, with S-NA15 suppositories releasing 98.6 (± 0.9) % and PEG suppositories releasing 94.9 (± 1.2) % amoxTH in the first 30 min. This was even more rapid than release from the commercially available oral capsules, which released 83.4 (± 8.7)% over the first 30 min (Fig. 3).

The presence of the HPMC outer shell in S-NA15_OEC_ resulted in a slower, steady release of amoxTH, with 89.9 ± 9.4% amoxTH release over 120 min. This is because the outer HPMC shell had to disintegrate in order to release its content. Further investigation is required to ascertain the effect of prolonged contact of the formulation with the rectal mucosa.

### Irritation potential

The results of the slug mucosal assay are presented in Table 3. The negative control (Novata B) did not induce irritation according to the classification prediction model, producing only 1.8 ± 0.2% mucus over 5 days, whereas the positive control Novata B with SLS induced severe irritation, with mucus production of 30.0 ± 1.8% and no slugs surviving to the end of the study period. This aligns with previous research, which found that Novata B induced no enzyme release and a low mucus production and protein release, whereas Novata B with 10% SLS induced enzyme and protein release as well as high mucus production [26, 27]. These findings validated the experimental conditions of the test system. For each formulation, irritation was slightly higher in medicated suppositories compared with their non-medicated equivalent. While amoxTH alone induced 2.7 ± 1.3% mucus over the study period and can be considered non-irritant according to the classification prediction model, there is likely to be a cumulative effect from different components in a formulation.

**Table 3:** Classification of each test compound according to the classification prediction model of the slug mucosal irritation assay, where total mucus production is presented as a percentage of body weight (mean ± SD, *n* = 3)

Formulation	Total mucus production (% body weight)	Survival on day 5 (%)	Irritation classification
Negative control (Novata B)	1.8 ± 0.2	100	Non-irritant
Positive control (Novata B + SLS)	30.0 ± 1.8	0	Severe
AmoxTH powder	2.7 ± 1.3	100	Non-irritant
Suppocire-NA15	2.0 ± 1.5	100	Non-irritant
PEG 1500/PEG 4000 (80/20)	7.1 ± 1.3	100	Mild
Suppocire-NA15 + amoxTH	2.7 ± 0.3	100	Non-irritant
PEG 1500/PEG 4000 (80/20) + amoxTH	10.2 ± 4.0	100	Mild

Suppocire NA-15 was classed as non-irritant, with both blank and medicated formulations inducing less than the 7% mucus production threshold over the study period. The hydrophilic PEG formulations (blank and medicated) resulted in a higher mucus production during each contact period, with a total mucus production over the study period between 7 and 12%. Therefore, the PEG formulations were classified as causing mild irritation.

The reliability of this assay to predict nasal irritation as well as stinging and burning sensations have been demonstrated for several commercially available nasal formulations [19, 34, 35]. This model has also been used to test tolerance of ocular [20, 36] and vaginal [37] formulations. The irritation potency of some commercially available formulations has been classed as mild and moderate based on mucus production. While this was supported by adverse event reporting in corresponding in vivo studies, the formulations were considered innocuous if minimal proteins and enzymes were released [38]. This highlights the need for further research comparing the present findings with in vivo data to validate the slug mucosal assay for rectal applications to confirm whether mild irritation might represent a barrier to further development. Methods to reduce irritation of the PEG formulations and consideration of the tolerability over the proposed five-day treatment period will be the focus of future research. Further, irritation may be reduced by moistening the suppository with water prior to administration [39] but more research is needed to support this.

### Antibacterial efficacy

**Fig. 4:**
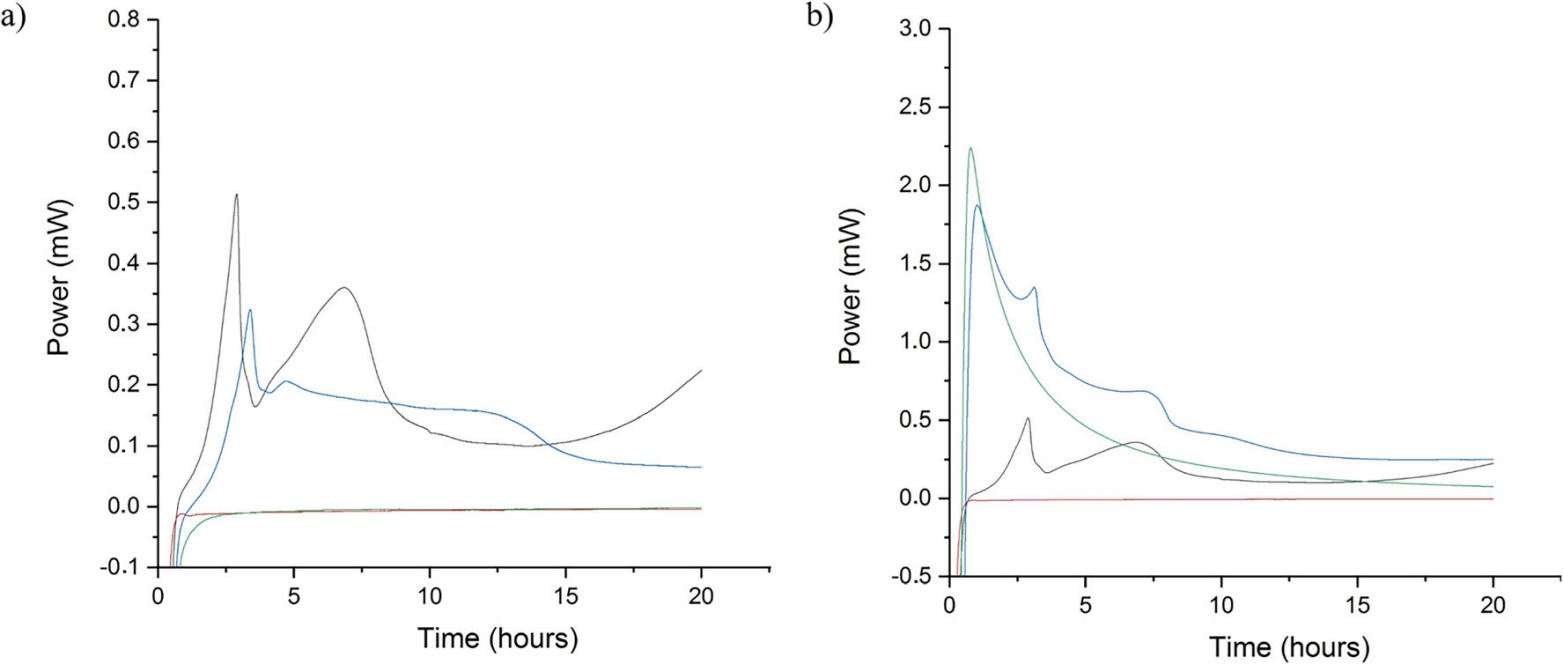
Calorimetric trace of *S. aureus* in nutrient broth in the presence of **a** S-NA15 suppositories and **b** PEG suppositories, showing power (mW) as a function of time for the blank control (black), amoxTH (red) blank suppositories (blue) and suppositories medicated with amoxTH (green)

Both S-NA15 and PEG suppository formulations showed antibacterial efficacy equivalent to pure amoxTH against *S. aureus* when tested with IMC and plate counts. Each of the frozen *S. aureus* aliquots had an average bacterial population of 10^6^ CFU/mL, and sterility was also confirmed. The calorimetric trace (power, mW, as a function of time, hours) of *S. aureus* in nutrient broth without amoxicillin present showed a biphasic exponential signal with two exothermic peaks in the first 8 h, followed by an almost flat signal, characteristic of *S. aureus* in nutrient broth (Fig. 4). The first exponential phase (at around 2.5 h) represented aerobic bacterial metabolism. When the limited amount of oxygen contained in the hermetically sealed ampoule was exhausted, organisms switched to anaerobic metabolism, represented by the second exponential phase (at around 6 h) [29]. Post-calorimetric bacterial enumeration revealed an *S. aureus* population of 10^7^ CFU/mL. When testing the antibacterial efficacy of a pure sample of amoxTH against *S. aureus* (positive control), no power signal was recorded via IMC, implying no bacterial growth. This was supported by the absence of bacterial growth on nutrient agar plates post-calorimetry. This confirmed the antimicrobial activity of amoxicillin under the experimental conditions described. With the formulated amoxicillin suppositories, S-NA15 completely melted during the 30-min equilibration period before data capture was initiated, implying the release of amoxTH prior to data capture (Fig. 4a). A similar scenario was observed for the blank S-NA15 suppositories. This could be the reason for the flat signal observed for the S-NA15 medicated suppositories throughout the entire duration of the assay. Post-calorimetric assay via plate count method on nutrient agar also revealed no bacterial growth. Blank S-NA15 suppositories did not inhibit bacterial growth, resulting in the biphasic exothermic peak of *S. aureus* as observed in the control. The MIC range for amoxicillin against Staphylococci is 0.03–128 μg/mL [40]; therefore, medicated suppositories containing 250 mg amoxicillin (as seen with the capsules) would be too concentrated for this test. Hence, to achieve a final concentration of 128 μg/mL amoxTH, medicated suppositories containing 1.920 mg amoxTH were prepared and transferred into ampoules containing 15 mL nutrient broth as described above.

When medicated suppositories were prepared with PEG, differences were observed in the power-time trace compared with the S-NA15 formulations. Unlike the S-NA15 formulations where we noted complete dissolution of suppository before data capture, the dissolution of PEG was incomplete before the beginning of data capture. This led to the recording of an exothermic peak in the calorimetric trace (Fig. 4b). Upon complete dissolution of the medicated PEG suppositories, a flat line was observed. This implied no power being generated with time and as such no bacterial metabolism and subsequent bacterial growth. Post-calorimetric enumeration via plate count on nutrient agar confirmed no bacterial growth. Blank PEG suppositories did not inhibit bacterial growth, resulting in a calorimetric trace comprising both the dissolution process and the biphasic exothermic *S. aureus* growth.

### Preliminary stability studies

There were no visual changes from time 0 in formulations stored for 2 months at 4 °C (refrigerated) or 23 °C/40%RH (ambient conditions). Both S-NA15 and PEG suppositories maintained their shape and initial physical appearance, although the content of the S-NA15 suppositories was about 15% lower after 2 months, suggesting that further protection is required to maintain acceptable stability (Table 4**)**. As expected, high-temperature conditions resulted in complete melting of S-NA15. The process of over-encapsulation with an HPMC shell appeared to improve stability of S-NA15 suppositories, with reduced degradation of amoxTH over time. This is likely because the HPMC shell acted as a physical barrier to the environment and also contained any of the melted/dissolved formulation. Preliminary studies showed that formulations stored for 2 months at 40 °C/75%RH in aluminium ziplock pouches underwent a high degree of degradation. On reflection, it was thought that humidity caused the degradation, with HPMC swelling and PEG dissolving in the high-moisture environment, causing complete loss of structure. For this reason, formulations stored at 40 °C were enclosed in sealed aluminium pouches. This offered higher protection against humidity compared with the aluminium ziplock pouches previously used. However, partial leakage of content was recorded both at 75% RH and 10% RH, at the same temperature conditions, showing that the high-moisture environment was not the only cause responsible for physical instability of encapsulated formulations. PEG suppositories underwent partial melting at 40 °C/75%RH, with more than 20% amoxTH degradation. Although the melting point of the PEG mixture was higher than 40 °C, suppositories most likely dissolved due to the high moisture environment (75% RH), a process further promoted by the hygroscopicity of PEG. This hypothesis was also confirmed by analysing PEG suppositories after storage for 2 months at 40 °C/10% RH, where no physical change from time 0 was recorded and only 16% degradation was measured.

**Table 4:** Changes in visual assessment and amoxicillin content (mean ± SD, *n* = 3) of selected formulations (S-NA15, S-NA15_OEC_ and PEG) from time 0 following two months storage at 4 °C (refrigerated), 23 °C/40% RH (ambient conditions), 40 °C/75%RH, 40 °C/10%RH. NT = not tested

Base	Assessment	Two-month storage condition			
		4 °C	23 °C/ 40%RH	40 °C/ 75%RH	40 °C/ 10%RH
S-NA15	Visual assessment after 2 months	No change	No change	Phase separation, melted, odour	Phase separation, melted, odour
	% Original content (±SD)	85.2 (5.8)	84.3 (3.6)	NT	NT
S-NA15_OEC_	Visual assessment after 2 months	No change	No change	Odour, change in colour	Visible leakage of content
	% Original content (±SD)	104.9 (2.3)	NT	109.7 (4.9)	94.9 (4.9)
PEG	Visual assessment after 2 months	No change	No change	Partial melting, odour	No change
	% Original content (±SD)	112.7 (1.5)	101.0 (7.7)	78.1 (2.2)	84.4 (5.9)

While over-encapsulation of the S-NA15 formulation showed promise in terms of stability over 2 months, further research is needed to optimise these formulations to offer extended stability and determine in vivo efficacy. There are a variety of different formulation strategies that have been investigated to offer better control over spreading, retention and drug release of rectal drug delivery systems [11, 41, 42]. Recently, rectodispersible tablets and capsules containing ceftriaxone have been developed in a proof-of-concept study. Formulations passed 6-month accelerated stability tests and, when administered to rabbits, resulted in ceftriaxone plasma concentrations greater than the MIC for over 2 hours [32]. Further research into the development of a rectodispersible amoxicillin tablet, similar to the oral dispersible tablet currently on the market, would be worthwhile. Further, 3D printing of rectal formulations may allow researchers to further optimise the properties of suppositories and develop formulations with predictable release profiles. Indeed, 3D printing of a water-soluble polymer suppository shell has been successfully developed to offer desirable drug release using progesterone as a model drug [43]. Research into the potential application of 3D printing for antimicrobial suppositories is justified.

## Conclusions

Access to medicines, including their availability and affordability, is a major public health challenge worldwide. In the present work, different suppository bases and their potential to reformulate 250 mg amoxicillin for the rectal route were explored. Lipophilic (S-NA15) and hydrophilic (PEG 80% PEG 1500/20% PEG 4000, w/w) commercial suppository bases with known safety and precedent of use were selected and both types of suppositories showed promising results when characterised in vitro. They offered rapid disintegration and similar drug release profiles to commercial amoxicillin capsules, with rapid and complete amoxicillin release within half an hour. An HPMC outer shell was used to enhance stability of S-NA15 suppositories at higher room temperature, but it also prolonged their disintegration and slowed down amoxicillin release. The slug mucosal assay demonstrated that S-NA15 suppositories were non-irritant, and PEG suppositories were only mildly irritant. Confirmation of these findings with in vivo studies will validate these findings and may lead to a simple in vitro screening test for future rectal formulations. Antibacterial activity was observed for both lipophilic and hydrophilic formulations against *S. aureus*. To leverage the need for cold chain, the rectal formulations developed in the present work containing PEG and over-encapsulated S-NA15 have shown promise based on both the described in vitro testing and the preliminary stability studies. Further in vivo testing concomitant to formulation development will allow this work to be contextualised towards a product with defined biopharmaceutical characteristics and commercial potential.
